# mHealth Technology Design and Evaluation for Early Childhood Health Promotion: Systematic Literature Review

**DOI:** 10.2196/37718

**Published:** 2022-10-06

**Authors:** Akeiylah DeWitt, Julie Kientz, Tumaini R Coker, Kendra Liljenquist

**Affiliations:** 1 Department of Human-Centered Design and Engineering University of Washington Seattle, WA United States; 2 Seattle Childrens Research Institute Seattle, WA United States; 3 Department of Pediatrics University of Washington Seattle, WA United States

**Keywords:** mobile health technologies, early childhood health promotion, child development, parent support technologies, mobile phone

## Abstract

**Background:**

Recent increases in smartphone ownership among underserved populations have inspired researchers in medicine, computing, and health informatics to design and evaluate mobile health (mHealth) interventions, specifically for those supporting child development and growth. Although these interventions demonstrate possible effectiveness at larger scales, few of these interventions are evaluated to address racial disparities and health equity, which are known factors that affect relevance, uptake, and adherence in target populations.

**Objective:**

In this study, we aimed to identify and document the current design and evaluation practices of mHealth technologies that promote early childhood health, with a specific focus on opportunities for those processes to address health disparities and health equity.

**Methods:**

We completed a systematic literature review of studies that design and evaluate mHealth interventions for early childhood health promotion. We then analyzed these studies to identify opportunities to address racial disparities in early- and late-stage processes and to understand the potential efficacy of these interventions.

**Results:**

Across the literature from medical, computing, and health informatics fields, we identified 15 articles that presented a design or evaluation of a parent-facing health intervention. We found that using mobile-based systems to deliver health interventions was generally well accepted by parents of children aged <5 years. We also found that, when measured, parenting knowledge of early childhood health topics and confidence to engage in health-promoting behaviors improved. Design and evaluation methods held internal consistency within disciplines (eg, experimental study designs were the most prevalent in medical literature, while computing researchers used user-centered design methods in computing fields). However, there is little consistency in design or evaluation methods across fields.

**Conclusions:**

To support more interventions with a comprehensive design and evaluation process, we recommend attention to design at the intervention (eg, reporting content sources) and system level; interdisciplinary collaboration in early childhood health intervention development can lead to large-scale deployment and success among populations.

**Trial Registration:**

PROSPERO CRD42022359797; https://tinyurl.com/586nx9a2

## Introduction

### Background

Early childhood health outcomes, such as social, motor, and cognitive development, largely depend on parental knowledge and behaviors. Both the American Academy of Pediatrics and the Centers for Disease Control provide guidelines for parents that educate them on health promotion strategies for their children [1,2]. These guidelines are often presented in local health centers, schools, or community sites [3]. However, finding and acting on information about early childhood health can be challenging [4,5]. For families affected by racial and economic disparities, having access to information, care providers, and resources to support health-promoting behaviors is a substantial barrier to parental action [6]. Mobile phone–based interventions have been developed to provide parents education on child health topics [7]. These interventions have been evaluated in highly diverse populations and are shown to be feasible for deployment at a larger scale, especially in lower-resource areas [8,9].

The Bright Futures guidelines from the American Academy of Pediatrics for early childhood health promotion outline three areas of focus for comprehensive child development practice: (1) anticipatory guidance, (2) development and behavior screening, and (3) social determinants of health screening. Anticipatory guidance topics refer to proactive advice on activities that promote healthy growth, including nutrition, dental care, and physical activity [1,10]. Development and behavior screening includes tracking and monitoring milestones such as motor and cognitive development, growth, and communication skills [11]. Screening for social determinants of health includes monitoring the environment in which the child grows, including topics such as parent smoking behavior, housing, food security, and parent social support networks [12]. Pediatric experts have referenced the importance of addressing all 3 topics in regular visits with pediatric patients to identify upstream factors that may affect development [13] and to understand the challenges of parents when adhering to recommendations. There is an opportunity to address the effects of health inequity on experiences with mobile health (mHealth) technologies [14,15].

### Objectives

This systematic literature review aimed to document current research on mobile-based health promotion interventions and understand the methods used to design and evaluate these systems. As we focused on parent-facing interventions for early childhood health (ages 0-5 years), we also examined the opportunities for design and evaluation in this area to critically engage with the potential for racial disparities in intervention effectiveness. In this study, we aim to answer these research questions:

1. What are the design, evaluation, and reporting practices in computing, medical, and health informatics fields for early childhood health interventions?
2. What opportunities exist to address the risk of technology-generated disparities in early childhood health interventions’ design, evaluation, and reporting practices?

### Prior Work

#### mHealth Interventions

mHealth interventions use mobile systems (including SMS text messaging, mobile apps, mobile-optimized websites, and wearable technologies) to deliver health interventions [16]. mHealth interventions are commonly developed and tested in low-income or middle-income communities [17]. They are described as providing fast access to care, being low cost to build and implement, and being accessible because most people own a cell phone. Researchers have explored opportunities for mHealth interventions to support both adults and children with self-management of their health [8,18]. Researchers have also developed interventions that support caregivers with monitoring the health of others [19].

mHealth interventions have the potential to extend health intervention content to hard-to-reach populations, they are often criticized for their lack of regulatory oversight, potential data privacy risks, and lack of implementation in clinical settings [20].

#### Intervention-Generated Disparities

Health disparities between groups occur when one group in a population experiences higher levels of poor health outcomes compared with the general population [5]. Both socioeconomic factors and health systems can influence access to resources that influence health outcomes [21]. Researchers have developed health equity models that address *upstream factors* [22], such as socioeconomic status, to identify the causes of disparity and adapt care to address those causes [22]. Although health interventions are designed to reduce poor health outcomes in specific groups, researchers have identified that considering health equity in designing and evaluating interventions is crucial to prevent intervention-generated inequalities [23]. Intervention-generated inequality occurs when interventions are more effective for already advantaged groups, widening the disparity between groups that are doing well and those that are not. Veinot et al [22] identified the characteristics of health interventions that worsen inequalities between disadvantaged and advantaged groups. In this work, they present a model to prevent intervention-generated inequalities by addressing inequality in access, uptake, adherence, and effectiveness and recommend prevention opportunities in the evaluation and reporting phases.

#### mHealth Literature Reviews

mHealth intervention research exists at the intersection of computing, health informatics, and medical disciplines, which are highly segmented and specialized. To identify trends across these fields, researchers have used the literature review method in many forms to survey existing research on mobile-based technologies and to examine opportunities for growth in the field. Berrouiguet et al [24] summarized the use of SMS text messaging as a health care tool for psychiatric disorders and reported evaluation methods and positive perceptions of SMS text messaging by participants. Lau et al [25] coupled a systematic search of mobile app stores with a literature review of psychosocial wellness. Bradway et al [26] used a scoping literature review to identify the qualitative and quantitative methods used to evaluate mHealth systems for chronic disease self-management and identified the best practices for comprehensive evaluations of complex mHealth tools. Wang et al [27] conducted a *systematic review of systematic reviews* to evaluate the potential of mHealth interventions to support diabetes and obesity treatment and management. Although mHealth interventions are promising, they identified that further research is needed to establish long-term effectiveness. Anderson-Lewis et al [28] also evaluated mHealth interventions deployed in historically underserved and minority populations in the United States and recommended that research should expand to include mobile phone and tablet apps. To our knowledge, there have been no systematic evaluations of mHealth interventions designed to support early childhood health or evaluations that focus on how racial disparities potentially influence the effectiveness of these interventions. Our review intends to survey the work happening in computing, medical, and health informatics fields to identify opportunities to address racial disparities in the evaluation and design of health interventions. We also intend to bridge findings across disciplines to promote the effectiveness of delivery systems, design methods, evaluation methods, and reporting standards that future interventions might adopt.

## Methods

### Reporting Standards

**Figure 1:**
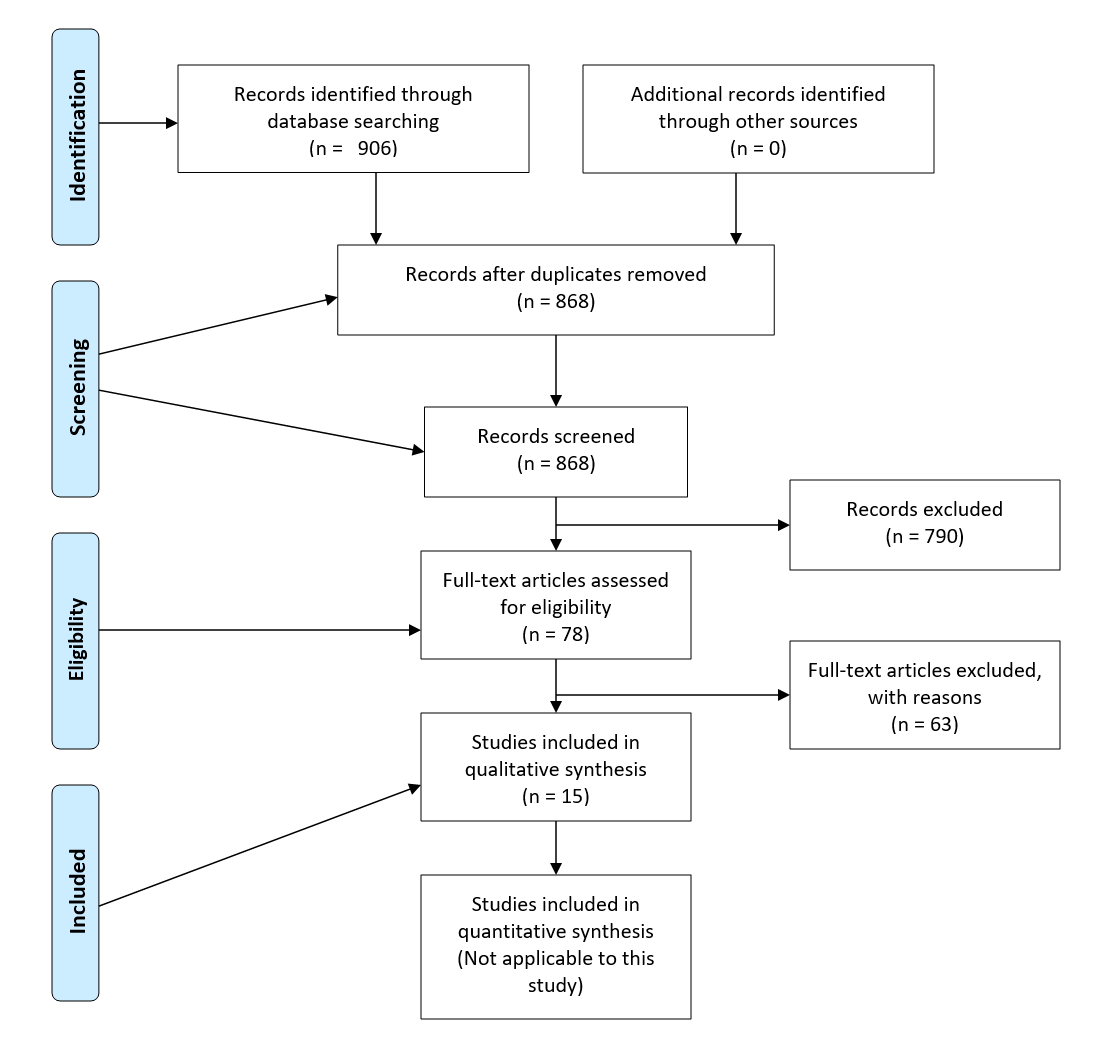
Flow diagram of the study selection process.

We completed the PRISMA (Preferred Reporting Items for Systematic Reviews and Meta-Analyses) checklist and confirm that the study is compliant. The full protocol for this study is available in Figure 1.

### Search Strategy

We completed a database search for full-text scholarly articles in medical, computing, and health informatics fields in February 2022 using the electronic databases PubMed, Embase, CINAHL Complete, ERIC, Compendex, Inspec, and ACM Digital Library. We coordinated with our university’s health sciences library to identify these databases, as they are relevant to medicine, technology, and research at the intersections of health and technology, where we would expect to find the literature on mobile-based health interventions.

Our search strings included terms describing early childhood health, mobile technologies, and the parents and primary caregivers of young children. We refined and adapted the keyword strings to be compatible with the unique search mechanics of each database (eg, using different typographic marks as search operators). The complete search strings by database are presented in Multimedia Appendix 1. We limited our search to studies within the past 10 years (2011 to 2022) to reflect the rapid rate at which technology development and adoption evolves [29].

### Selection Criteria

We included studies if they (1) presented and tested a mobile app, SMS text messaging system, or mobile website to be used by participants; (2) included a health scope related to anticipatory guidance, development and behavior screenings, or social determinants of health topic areas outlined in Bright Futures Guidelines for Health Supervision of Infants, Children, and Adolescents, fourth edition; (3) targeted parents or guardians of children aged 0 to 5 years directly as users; (4) included a study related to the practicality of the app for target users (eg, usability, feasibility, pilot study, or randomized controlled trial); (5) were published within the past 10 years; and (6) are a completed, peer-reviewed journal paper or conference paper.

Studies were excluded if they (1) involved a study of a mobile app created to support pregnancy or postpartum health alone, (2) exclusively targeted other caregivers as end users for the system (eg, day care providers, paid caregivers, nurses, and community health workers), or (3) consisted solely of randomized controlled trial protocol documentation. In addition, we excluded studies not written in English, government reports, articles, and opinion pieces.

### Selection Process

The database search results were downloaded and organized in a spreadsheet and duplicates were removed. One researcher screened the search results by using the inclusion and exclusion criteria in 3 distinct groupings. First, we used the inclusion and exclusion criteria to screen the titles of the results. Next, we accessed the abstracts for the remaining results and applied the inclusion and exclusion criteria. Finally, we performed a full-text review of the remaining studies. The PRISMA flow diagram detailing the number of studies present in and after each phase is presented in Figure 1.

### Data Extraction

One researcher reviewed each full text of the included studies and documented the relevant information in a spreadsheet. This information included (1) titles, authors, country, and year of publication; (2) type of field the study was published in (eg, computing, medical, and health informatics); (3) type of mobile technology the study evaluated (eg, texting or SMS text messaging system or mobile app); (4) study design used to evaluate the technology; (5) target population; (6) number of participants recruited for the study and their reported demographics; (7) features and functionalities of the mobile technology; (8) sources for content in the mobile technology; (9) outcomes measured for the child; (10) reported parent perceptions of the technology and outcomes related to changes in parent knowledge and decision-making processes; and (11) reported outcomes for usability, feasibility, or acceptability.

## Results

### Selection and Inclusion of Studies

We screened 906 results from database searches and excluded 891 (98.3%) studies during the screening process. We removed 38 duplicates before beginning the screening process. During title screening, we excluded 83.3% (755/906) of studies. Of the remaining 151 studies, we excluded 73 (48.3%) studies during the abstract screening phase, leaving 78 (51.7%) papers for full-text screening. We excluded 6.9% (63/906) of studies during the full-text screening process, leaving 1.7% (15/906) studies that met the inclusion criteria. Figure 1 visually represents the number of studies excluded during each phase of the screening process.

### Characteristics of the Included Studies

The full overview and characteristics of the studies are presented in Table 1. The publication dates ranged from 2014 to 2021, and most studies (9/15, 60%) were published in 2017, 2019, or 2020. Among the 15 studies, 11 (73%) were published in journals and 4 (27%) were peer-reviewed full conference papers.

All (15/15, 100%) the studies developed and contributed to a novel intervention. Overall, 7% (1/15) of studies evaluated an existing mobile app and iterated its design with feedback from parents [30]. Of 15 studies, 3 (20%) studies evaluated only the feasibility of the intervention [32,34,35], whereas 8 (53%) studies evaluated the intervention’s potential to achieve specific health outcomes [36-39,41-44]. The technologies evaluated in these studies included 8 mobile apps [30,33,35,38,40-43], 4 SMS text message systems [32,36,37,44], 1 voice message system [34], 1 website optimized for mobile devices [39], and 1 social media platform [31]. A total of 40% (6/15) of articles reported technical specifications for how they built and deployed the intervention [31,32,36,37,41,43], 40% (6/15) of studies were conducted in the United States [31-33,38-40], and 6% (1/15) of studies was dually conducted in the United States and Mexico [30]. Overall, 20% (3/15) of studies were conducted in Iran [37,42,43], and the remaining (5/15, 33%) studies were conducted in Cambodia [34], China [36], Guatemala [44], Sweden [41], and Switzerland [35]. Tables 2 and 3 provide detailed information about the study findings and technologies evaluated.

**Table 1:** Article characteristics.

Study	Field	Country	Number of Participants (parents)	ORBIT^a^ model classification
Armenta et al [30], 2019	Computing	United States and Mexico	11	Nonexperimental evaluation of feasibility; no measurement or documentation of child health outcomes
Suh et al [31], 2014	Computing	United States	14	Nonexperimental evaluation of feasibility; no measurement or documentation of child health outcomes
Olson et al [32], 2016	Medical	United States	31	Nonexperimental evaluation of feasibility; no measurement or documentation of child health outcomes
Hayes et al [33], 2014	Computing	United States	14	Nonexperimental evaluation of feasibility; no measurement or documentation of child health outcomes
Huang and Li [34], 2017	Medical	Cambodia	126	Nonexperimental evaluation of feasibility; no measurement or documentation of child health outcomes
Jacques et al [35], 2020	Health informatics	Switzerland	12	Nonexperimental evaluation of feasibility; no measurement or documentation of child health outcomes
Jiang et al [36], 2019	Health informatics	China	558	Nonexperimental evaluation of feasibility; no measurement or documentation of child health outcomes
Khademian et al [37], 2020	Medical	Iran	211	Pilot and early experimental evaluation of child health outcomes
Lozoya et al [38], 2019	Medical	United States	33	Pilot and early experimental evaluation of child health outcomes
Nezami et al [39], 2018	Pediatrics	United States	51	Pilot and early experimental evaluation of child health outcomes
Nolen et al [40], 2018	Health informatics	United States	8	Pilot and early experimental evaluation of child health outcomes
Nystrom et al [41], 2017	Medical	Sweden	315	Pilot and early experimental evaluation of child health outcomes
Seyyedi et al [42], 2020	Medical	Iran	110	Pilot and early experimental evaluation of child health outcomes
Zolfaghari et al [43], 2021	Medical	Iran	58	Pilot and early experimental evaluation of child health outcomes
Domek et al [44], 2016	Medical	Guatemala	321	Pilot and early experimental evaluation of child health outcomes

^a^ORBIT: Obesity-Related Behavioral Intervention Trials. The ORBIT model establishes a pathway of phases that supports the translation of information in behavioral and social science research into health interventions [45].

**Table 2:** Summary of findings.

Study	Technology description	Study design	Usability and feasibility evaluations of the technology.	Child outcomes	Parent knowledge and decision-making
Armenta et al [30], 2019	Mobile app for child milestone tracking	Qualitative usability study—evaluated 3 versions of a mobile app: original, translation, and redesign	Evaluated the user interface and workflows for basic functions for the first app to identify objectives for a redesign. Found that the first app had several issues with basic functions (eg, data entry and creating new profiles). Evaluated the redesigned app and successfully resolved usability issues previously identified.	Not measured	Not measured
Suh et al [31], 2014	Social media network (Twitter), website, and text messaging system for tracking child health milestones	Deployment study and qualitative, exploratory study	Parents reported difficulty with responding to tweets using the program’s syntax and did not like that the program used a social networking site. Parents liked the accessibility of the content related to child milestones and opportunities to interact with other parents through the platform.	Not measured	Not measured
Olson et al [32], 2016	SMS texting with personalized messages about child development and local child health resources	Feasibility study	Parents reported high satisfaction with the frequency of text messages. Parents also shared preference for text messages over website-based programs, owing to ease of access.	Not measured	Parents reported increased awareness of language-promoting activities and local child development resources
Hayes et al [33], 2014	Mobile app for tracking infant weight, diapers, infant emotions, reminders, and parent moods	Qualitative technology probe, interviews, surveys, and log analysis	Did not track any usability issues. Parent feedback revealed that the app does not require much training to use it as a beginner.	Not measured	Parents expressed that the app supported parent-focused outcomes (tracking mental health) and that using the app did not contribute to additional stress levels
Huang and Li [34], 2017	Interactive voice response system by using prerecorded voice phone calls	Feasibility study	Intervention was well accepted by parents, as parents expressed interest in paying for the service and referenced the tool’s cultural relevance.	Not measured	Not measured
Jacques et al [35], 2020	Mobile app for recording food quality and intake and tracking nutrition information of foods	Feasibility study	Parents rated the app as high on the ease-of-use scale [46].	Not measured	Not measured
Jiang et al [36], 2019	SMS texting with information about feeding and breastfeeding	Quasi-experimental design	Not measured	Measured child’s BMI before and after intervention. Intervention did not demonstrate a significant effect on the children’s BMI	Not measured
Khademian et al [37], 2020	SMS texting with information about child oral health	Randomized control trial	Not measured	Not measured	Maternal knowledge about oral health and related practices improved after intervention
Lozoya et al [38], 2019	Mobile app with guided videos, reminders, and social feed for child’s oral hygiene	Experimental pretest-posttest and qualitative interviews	Not measured.	Documented dietary habits, oral health practices, and dental appointment attendance for all children before intervention. Did not find any changes to those practices after intervention	Did not find a significant quantitative change in parent knowledge. Found that parents reported a positive experience with the mobile app’s reminders and guided brushing features
Nezami et al [39], 2018	Mobile-optimized website, SMS text messages, and physical list of foods with nutrition information	Randomized controlled trial	Adherence to the intervention was higher than in previous studies with mothers of young children. Dropout was more likely among people of color; however, dropout did not differ by treatment group.	Children consumed less beverages in the intervention group	Not measured
Nolen et al [40], 2018	Mobile app with videos, reminders, and facts about a child’s oral health	Usability study	On average, parents believed that the app could keep them informed about their child’s oral health. Parents rated navigation of the interface and design elements as poor. Parents shared that several of the features in the app did not work.	Not measured	Not measured
Nystrom et al [41], 2017	Mobile app for tracking child’s food intake and exercise	Randomized controlled trial	Not measured	Measured child BMI or FMI^a^ levels and did not find a change after intervention. Found that child activity levels increased	Not measured
Seyyedi et al [42], 2020	Mobile app with guidance on feeding and direct chat with clinicians	Randomized controlled trial	Not measured.	Intervention group improved nourishment status	Mother’s nutritional literacy improved for both groups; however the intervention group had greater improvement
Zolfaghari et al [43], 2021	Gamified mobile app with tracking and reminders for oral hygiene practices	Pretest-posttest controlled clinical trial	Not measured.	Reported significant improvement in child tooth brushing frequency. Both groups had reduced child plaque measurements, but reduction was higher in the gamified intervention group	Measured improvements in parent knowledge about oral health in both groups, but higher improvement was found in the gamified group
Domek et al [44], 2016	Vaccine reminder texting program	Pilot randomized controlled trial	Identified that the vaccine SMS texting reminder system is feasible for the LMIC^b^ context, and reported high user satisfaction with the technology.	No significant impact on vaccine rates in the intervention group compared with the control group	Parents expressed that the reminders were helpful in following up with their child’s vaccine series

^a^FMI: fat mass index.

^b^LMIC: lower middle–income country.

**Table 3:** Technology systems and features.

Studies	Technology system	Functions and features	Early childhood areas (as outlined by Hagan et al [1])	Content sources
Armenta et al [30], 2019	Smartphone app	Translated version of existing smartphone app (from English to Spanish). Includes developmental milestone tracking through checklists, exporting, and sharing completed checklists, and recording notes about milestones. Supports profiles for >1 child.	Developmental milestone surveillance	First iteration of mobile app developed using the CDC’s^a^ Learn the Signs. Act Early campaign. The second iteration of the mobile app was derived from the Spanish version of the CDC’s milestone list
Suh et al [31], 2014	Social media network (Twitter), website, and SMS text messaging	Parents follow an account that shares age-based milestone questions (sometimes coupled with images) at regular intervals. Then, the parent can respond by posting a tweet or direct messaging the account.	Developmental milestone surveillance	Not reported
Olson et al [32], 2016	SMS text messaging	Sends 3 SMS text messages per week for 12 weeks with information on child development and local child health resources. Sends messages with survey questions about parent’s strategies to support their child’s health.	Developmental milestone surveillance	Not reported
Hayes et al [33], 2014	Smartphone app	Tracking infant weight, diapers, and emotions. Includes mood tracking for parents. Generates data files for health care professionals and reminders for tracking in the app.	Feeding, growth development, and parent mental health	Not reported
Huang and Li [34], 2017	Interactive voice response system	Sends prerecorded messages through phone call to parents, starting 3 days after birth. Messages are sent every 4 days until the child is 28 days old. Messages are 60-90-seconds long and have a variety of voices offered.	Developmental milestone surveillance	Consulted with local midwives for more information about message content
Jacques et al [35], 2020	Smartphone app	Digitizes food recording features, including intake and quantity. Provides information on added fats or sugars in foods after parents use the app to take pictures of food labels.	Food and nutrition	Consulted with expert pediatric dietetics at Geneva Children’s Hospital
Jiang et al [36], 2019	SMS text messaging	Weekly text messages provide anticipatory guidance about feeding, and requests more information from parents about breastfeeding statuses for themselves and their child.	Feeding and breastfeeding	Developed using WHO^b^ breastfeeding and infant or young child feeding recommendations. Consulted with local child health care experts
Khademian et al [37], 2020	SMS text messaging	Daily SMS text messages provide guidance about oral health. SMS text messages were designed using gain- and loss-frame formatting.	Care of teeth and gums	Consulted with local pediatric dentistry professors and educational management specialists
Lozoya et al [38], 2019	Smartphone app	Provides documents and videos with oral hygiene instructions. Tracks tooth brushing times and sends brushing reminders. Includes a social feed to share brushing and flossing experiences with a social network.	Care of teeth and gums	Not reported in this paper; documented in preceding paper
Nezami et al [39], 2018	Mobile-optimized website, SMS text messaging, paper-based list, stickers, and charts	Text message prompt at the end of every week to collect the mother’s personal data, which is then used to create a tailored email about nutrition and quality of foods consumed.	Food and nutrition	Not reported in this paper; documented in a preceding protocol paper
Nolen et al [40], 2018	Smartphone app	Sends tooth brushing reminders for morning and night, tracks frequency of brushing and flossing events, includes videos for guided brushing, and has facts about oral health in articles.	Care of teeth and gums	American Dental Association website
Nystrom et al [41], 2017	Smartphone app	Mobile app sends push notifications with general information about nutrition and exercise. Provides advice and strategies to change behaviors, supports weekly tracking of child’s intake and exercise. App provides weekly feedback (graphical and automated comments) based on personal data. The mobile app also supports direct contact with a dietician or psychologist.	Food and nutrition, physical activity	Not reported
Seyyedi et al [42], 2020	Smartphone app	Provides articles with age-based guidance education based on feeding children. Provides a chat feature where clinicians can directly answer parent questions in the app.	Feeding and breastfeeding	Maternity Guidelines for Maternal and Child Health Services issued by the Iranian Ministry of Health. Cross-referenced content with guidance from a local nutritionist
Zolfaghari et al [43], 2021	Smartphone app	Provides written information about oral hygiene, nutrition, fluoride intake, and content of dental visits. Mobile app sends reminders to brush teeth at night.	Care of teeth and gums	American Association for Pediatric Dentistry Guidelines. Mobile app was evaluated by oral medicine specialists, pediatric dentists, and electronic learning and programing technicians
Domek et al [44], 2016	SMS text messaging	SMS text message reminders sent to parents at 6, 4, and 2 days before the next scheduled child vaccination date (as part of a 3-dose vaccination series).	Vaccines	Guatemala Ministry of Public Health and Social Assistance, Pan American Health Organization, and project optimize

^a^CDC: Centers for Disease Control and Prevention.

^b^WHO: World Health Organization.

### Features of the Technology Interventions

Among the studies that evaluated mobile apps, features included a tracking component for parent and child behaviors, articles about child health topics, reminder systems using push notifications [33,38,40,43], milestone questionnaires [30], and data file generation for a physician to review [33]. SMS text messaging interventions provide anticipatory guidance for parents to save and review their child’s health and development [32,36], send reminders for in-person appointments [44], and request information about parent or child behavior status [36]. One intervention used the social media network Twitter, where parents would send tweets as responses to daily milestone questions [31]. Another intervention sent parents prerecorded phone calls with information about milestones multiple days per week for a month [34]. One intervention also provided personalized summaries of the tracked content to parents by email [39].

### Methods Used for Design and Evaluation

Studies from medical fields have generally used experimental methods to evaluate the feasibility or effectiveness of interventions. Of the 15 studies, 5 (33%) used randomized controlled trials [37,39,41,42,44], 2 (13%) used a pretest-posttest design [38,43], and 1 (6%) engaged parents in qualitative interviews to hear their experiences [38]. Moreover, 13% (2/15) of studies published in medical fields used a feasibility study to evaluate their intervention [32,34]. Studies published in computing fields have used methods from design disciplines to evaluate interventions. Furthermore, 13% (2/15) of studies asked participants to adopt the intervention in their everyday lives to understand its feasibility and acceptability. Of the 15 studies, 1 (6%) evaluation used a deployment study coupled with qualitative interviews [31], and the other used a technology probe and interviews, surveys, and a log analysis in their comprehensive evaluation [33]. The other computing study conducted a usability evaluation of their designs [30]. Studies published in health informatics fields have used interdisciplinary methods based on traditional computing and medical research. Of the 15 studies, 1 (6%) study experimentally measured changes in child weight and activity levels after the onset of the intervention [36], 1 (6%) study conducted a feasibility evaluation [35], and 1 (6%) acquired parent feedback through a usability study [40].

### Content Sources

A total of 20% (3/15) of studies from computing fields evaluated an intervention that supported parents in developmental milestone tracking [30,31,33]. Of these 3 studies, only 1 (33%) [30] mentioned its content sources for developmental milestone topics and related Spanish translations; however, another study referenced developing the intervention “based on a series of formative studies” [33]. Overall, 33% (1/3) of studies provided generic guidance for infants up to 28 days old and reported that they consulted local midwives for guidance [34]. The remaining studies addressed single-topic areas of early childhood health promotion.

Moreover, 26% (4/15) of studies focused on feeding- and nutrition-related content, 50% (2/4) of these studies were published in health informatics fields, and the remaining (2/4, 50%) studies were published in medical fields. Of these feeding and nutrition studies, 75% (3/4) reported how they developed the content for their intervention [35,36,42] and 50% (2/4) studies [36,42] consulted both national guidelines for feeding and nutrition and relevant experts (pediatric dietitians or nutritionists). Of these 4 studies, 1 (25%) study consulted pediatric dieticians at a local hospital where they were recruited for their study [35] and 1 (6%) study redirected attention to their related protocol paper for details on how they developed the intervention [39].

Overall, 26% (4/15) of studies presented an intervention targeting pediatric oral health and related parenting behaviors, and of these, 4 studies, 3 (75%) were published in medical fields [37,38,43]. Of these 3 studies, 1 (33%) reported that they reviewed national guidelines for pediatric dentistry and had their system evaluated by oral medicine specialists, pediatric dentists, and electronic learning and programing technicians [43]. The other (1/3, 33%) study reported that they consulted pediatric dentistry professors and an education management specialist to develop content for their intervention [37]. Furthermore, 33% (1/3) of studies did not report how they developed the content for the intervention [38]. The remaining pediatric oral health study was published in a health informatics field, and the intervention was developed using the American Dental Association’s website [40].

Of the 15 studies, 1 (6%) study targeted vaccine adherence and consulted the country’s Ministry of Public Health and Social Assistance, a health organization, and a special government project group focusing on vaccine adherence [44] and 1 (6%) study, which evaluated a speech- and language-focused intervention, did not report how they developed content for their intervention [32]. None of the studies in this review evaluated an intervention that comprehensively addressed anticipatory guidance, development and behavior screening, and social determinants of health topics, as recommended in the Bright Futures Guidelines for Pediatricians [1].

### Demographics Reporting

Across all studies in this review, the number of adult participants enrolled in the study ranged from 8 to 58. The demographics-reporting formats varied across all studies; however, all studies included similar demographic characteristics. Studies published in medical and computing fields reported at least three of the following characteristics: child age and gender, parent age and gender, income level, parent education level, mobile phone ownership or familiarity, and race or ethnicity characteristics. None of the studies published in health informatics fields reported race or ethnicity data of their participant samples. A total of 26% (4/15) of studies opted for nontraditional approaches to describe socioeconomic status: 1 (25%) study reported parental eligibility for a low-income support program [32], 1 (25%) reported parental use of rental accommodations [36], 1 (25%) reported parental work status [44], and 1 (25%) reported parents’ home or car ownership [42]. Moreover, 20% (3/15) studies that examined feeding or nutritional outcomes also tracked child weight or BMI [36,39,41].

### Feasibility of Mobile-Based Interventions for Parents and Children by Publishing Fields

#### Computing Fields

Evaluation objectives varied across the studies. More than half (8/15, 53%) of the studies in this review did not report changes in parents’ knowledge or decision-making processes [30,31,34-36,39-41]. Among the studies published in computing fields, 33% (1/3) of studies experimentally measured stress levels before and after the intervention and found that the intervention did not contribute to increased stress levels [33]. The same study found that their intervention scored high in their usability evaluations; parents reported ease of use during the onboarding process, and they appreciated seeing visualizations and parent-focused content (eg, information about parents’ mental health). Of the 3 studies, the other 2 (66%) published in computing fields did not report on outcomes related to parent or child behavior changes, as they focused on usability evaluations [30,31] and 1 (33%) study reported that parents had difficulty with the delivery system of the intervention through Twitter, mentioning that syntax made the response process difficult, and parents did not like sharing their child’s health information on a social network [31]. However, the same study also reported that parents generally appreciated the accessibility of content in the intervention. The other study reported that parents struggled during interface testing, as discovery of new features (eg, tracking milestones or creating a new profile) and related workflows were self-led, leading to parents perceiving the app as confusing and undirected [30]. The same study reported that parents preferred the ability to customize milestones that they share, increasing font size, and reviewing translations to Spanish, as they were not culturally relevant.

#### Health Informatics Fields

One interface-focused evaluation published in a health informatics field measured the intervention’s impact on child BMI, which demonstrated that it did not significantly impact the BMIs of children in the study [36]. Another study examining the usability of their gamified mobile app found that parents believed the app could keep them informed about their child’s oral health and support progress toward positive oral health behaviors [43]. The same study found that parents thought the app was user-friendly, although the interface design and process for parents to recognize and correct errors in tracking were rated low. This study also found that the gamified intervention was more effective in reducing child plaque than the nongamified approach. The remaining mobile apps published in a health informatics field reported a high ease of use of the interface and camera although parents had problems navigating the mobile app and expressed dissatisfaction with features that did not work [35]. However, the content, information, and reminders provided were rated as positive features in this app.

#### Medical Fields

Overall, 25% (2/8) of studies published in medical fields did not measure child-centered health outcomes [32,37]. These studies focused on changes in parenting behaviors or knowledge after the onset of the intervention or the feasibility of the intervention for evaluations in larger populations. In all, 12% (1/8) of studies found that maternal knowledge about pediatric oral health and related practices improved after the onset of the intervention and that high participation rates in the intervention indicated positive parent experiences with the technology [37]. In this intervention, parents specifically referenced that they liked the reminders and guided brushing videos the app provided. The other study reported that parents had increased awareness of language-promoting activities and local resources for child development support [32]. This same study reported that parenting behaviors that promote language development increased, and parents reported that the number of texts and content of the messages were accessible and easier to navigate than when searching the internet. Of 8 studies, 1 (12%) study did not evaluate interventions related to child outcomes or parent knowledge [34].

The remaining (4/8, 50%) studies published in medical fields measured child health outcomes after the onset of the intervention. Several studies have indicated that mobile-based interventions lead to significant child outcomes. Of the 8 studies, 1 (12%) study found that although BMI measurements of the intervention group did not differ significantly from those of the control group, physical activity levels did improve [41] and 2 (25%) interventions targeting nutrition-related outcomes, including reduced sugary beverage consumption [39] and improved child weight [42], found that children met the goals set during the intervention evaluation. Another study found a significant improvement in child toothbrushing frequency, and the gamified version of the intervention was more successful in controlling plaque than the control group [43]. However, 12% (1/8) of studies reported that the intervention had no significant impact on quantified child outcomes [38], despite positive experiences reported by parents.

## Discussion

### Principal Findings

We completed a systematic literature review of mobile-based health interventions for early childhood health promotion published within the past 10 years. Of the 15 articles we reviewed, we found that using mobile-based systems to deliver health interventions was generally well accepted by parents of children <5 years of age. We also found that, when measured, parenting knowledge of early childhood health topics and confidence to engage in health-promoting behaviors improved. For child health outcomes, several studies reported that the intervention did lead to targeted outcomes in child health, which indicates the potential for population-level improvements. In this section, we describe the opportunities for intervention designers and evaluators to critically engage with concepts in design practice, risk of technology-generated disparities, and reporting standardization.

### Progression of Research Studies

The Obesity-Related Behavioral Intervention Trials model establishes a pathway of phases that supports the translation of information in behavioral and social science research into health interventions [45]. Using the Obesity-Related Behavioral Intervention Trials model, we documented the preparedness of the systems evaluated in the studies for large-scale phase 3 efficacy testing. In phase 3 efficacy testing or clinical research, researchers examine the efficacy of interventions and monitor outcomes in larger, more diverse populations, and over longer periods. We identified that 53% (8/15) of the studies evaluated their systems using nonexperimental methods and established the feasibility of the systems for target populations without documenting child health outcomes [30-35,37,40]. The remaining (7/15, 46%) studies conducted early experimental evaluations of the systems in larger populations and evaluated related child outcomes [36,38,39,41-44]. However, it is important to note that of the 6 studies that completed large-scale evaluations, 83% (5/6) of studies were published in medical fields [37,39,41,42,44], and the other was published in a health informatics field [36]. This indicates a lack of large-scale efficacy evaluations of early childhood health technologies in computing and health informatics fields.

Computing researchers have identified that novel technology designs often do not reach larger-scale testing and deployment in larger populations owing to funding constraints, lack of organizational support to maintain systems, retention of designers at original organizations, and incompatibility between early-stage designs and large-scale clinical evaluation processes [47]. Multidisciplinary collaboration across computing, medicine, and health informatics can lead to larger-scale evaluations, as medical trials are more likely to be funded in the long-term [7]. Partnerships between these disciplines can also support higher-quality designs and evaluations as researchers can be dedicated to 1 area of a project. For example, the Text4Baby program included a multiyear collaboration between computing and medical researchers. This project led to evaluations specifically for low-income parents and was evaluated at multiple stages, including a pilot evaluation [48] and a randomized controlled trial [49]. The Text4Baby program was also evaluated across diverse contexts, including Spanish-speaking parents [48], pregnant people who smoke, and pregnant and postpartum people from underserved areas [50]. Chandler et al [51] documented the cultural tailoring practices for mHealth tools aimed at addressing sexual and reproductive health outcomes for black and Latina women and identified opportunities to improve long-term outcomes and address health disparities. In domains other than child development support, researchers have called for more impactful collaborations between computing and medical researchers. Calvo et al [52] documented an initiative to bridge researchers in computing, medicine, and health informatics around the global mental health epidemic and identified challenges and solutions related to interdisciplinary collaboration. As the applications of technology-based interventions for child development are often novel, there is an opportunity to recognize the success of interdisciplinary collaboration in other domains and set standards for future work in this area. With support across these disciplines, the early stages of the design and evaluation process can include larger and more diverse populations and introduce multiple dimensions of evaluation that address interface design, population relevance, and clinical objectives.

### Reporting Guidelines

We identified that there is inconsistency in the reporting of race or ethnicity data and socioeconomic backgrounds in the samples. Several studies in this review did not report the racial or ethnic backgrounds of the participants in their samples. In all, 33% (5/15) of studies did not report socioeconomic data for their participant samples [31,34,35,38,41]. Researchers have found that reporting the demographic makeup of research samples helps illuminate potential disparities in the effectiveness of novel systems [14]. To address the potential of interventions to contribute to intervention-generated inequality, Veinot et al [22] recommended setting recruiting objectives that lead to testing in more diverse samples by targeting members of both disadvantaged and advantaged groups in early evaluations. We also identified that there is consistency in demographic reporting formats within fields but not across them. To improve the generalizability of results across fields, researchers might rely on national guidelines for reporting demographics [53]. In addition, Siek et al [54] documented that certain racial disparities within technology use can sometimes be flattened when differences between groups are not reported or analyzed. Therefore, consistency in the reporting formats for racial demographics is necessary. Reporting demographics can also support broader research objectives to identify trends in technology use among specific populations [22]. As such, there is a need for researchers to both report their participant demographics with more granularity consistency and document the effectiveness of systems with attention to the unique experiences of different racial groups. Improvements in reporting have the potential to support more accurate and granular identification of those affected most by health disparities. For example, researchers have identified standards for demographic reporting that support the accurate identification of health disparities within public policy [55].

### Research Across Fields

The research objectives, methods, and paper formats tended to be consistent within fields. Among studies from medical fields, papers tended to be shorter in page length, focused on evaluating child health outcomes, and used quantitative methods to experimentally evaluate the effectiveness of the systems. Computing fields focused on using qualitative research methods to identify whether the design of systems was feasible for target populations and documented the opinions of participants on interface and interaction experiences. As expected, studies published in health informatics fields use a hybrid of methods from both computing and medical traditions, experimentally documenting child health outcomes and the feasibility of systems for deployment in larger populations. Researchers in computing, health informatics, and medical fields have all focused on the impact of usability and feasibility on the long-term effectiveness of interventions [23,56]. Researchers at the individual level might adopt a mix of qualitative and quantitative methods to complete more comprehensive evaluations of systems; however, interdisciplinary collaboration is needed to develop comprehensive and large-scale evaluations [54]. Partnerships between computing, medical, and health informatics researchers could lead to funding for large and long-term evaluations, a more comprehensive design process, and resources designated to developing content that addresses >1 need in the target population.

### Content Development Process Reporting

Reporting content sources support the decision-making process in uptake for both parents and pediatricians [4]. For pediatricians to recommend mHealth systems such that their guidance is aligned with the guidance from the systems, interventions should report their content sources and refer to national guidelines for content [12]. As mentioned in the studies from this review, an expert review of the content can be helpful in the design process. Although each study contributed a technology on a different topic area in child health (eg, some addressed nutrition, others addressed physical activity), none of the studies in this review developed a technology that comprehensively addressed anticipatory guidance, development and behavior screening, or social determinants of health topics.

The social determinants of health topics are of particular importance, as they have the potential to support communities affected by racial disparities. The impact of social determinants on health content is 2-fold. First, screening for social determinants of health can illuminate the health risk factors that are directly influenced by social contexts. Garg, Boynton-Jarrett, and Dworkin maintain that social determinants of health screening are imperative for identifying how race influences health outcomes [12]. Within child health promotion, social determinants of health screening can lead to tailored recommendations [13]. Second, the social determinants of health frameworks can be useful for informing the content of health technologies through features that are adjacent to core health guidance. For example, researchers have evaluated consumer health apps and have identified that the technology literacy, price, and system demands of mobile apps influence the user experience [57], which are all related to the social contexts in which people interact with systems. Thus, social determinants of health content can be relevant to both the content and implementation formats of technology systems.

### Design and Implementation Recommendations

There are several design, evaluation, and implementation recommendations that arise from the findings of this review and align with guidance in avoiding potential intervention-generated inequalities. Researchers might engage more diverse populations in the early design phases of systems to identify potential barriers to adherence in later testing phases and access them in later implementation phases. Computing researchers have identified that using human-centered methodologies in the early design and evaluation phases of system development leads to more effective and sustainable outcomes [58,59]. Including and reporting both the experiences of diverse populations and demographic sample makeup can illuminate potential disparities in health interventions. In this review, most studies focused on the evaluation of developed prototypes and sought to understand how to improve these designs for later iterations in the target populations. Although usability and feasibility evaluations are beneficial for determining goals for future designs, understanding the broader contexts in which people use systems requires further specificity [23]. Evaluating systems, including specific objectives to address the effectiveness of racially diverse communities, can promote the recognition of racial disparities. For example, Brewer et al [60] presented several case studies documenting the impact of context-specific considerations in health informatics interventions related to race and community. The case studies included in this work highlight strategies for implementation and design that directly respond to the experiences marginalized communities have with their health and related technologies. Unless there are specific objectives for late-stage evaluations to capture the experiences of underserved populations, these evaluations cannot respond to technology-generated disparities.

Involving underserved populations in early-stage design processes can illuminate the influence of racial disparities and the potential for technology-generated disparities. There is an opportunity to document the earlier stages of design and use methods in early-stage processes that promote meaningful engagement with the target populations. For example, researchers have relied on design methods that enable target populations to become cocreators of systems, including co-design [59] and participatory design [61]. There is a broad spectrum of participation in target populations, extending from the community level to individualized participation [62]. Early-stage involvement in design processes is crucial to meaningfully address the risk of technology-generated disparities, as design specifications born out of conversations with target populations can respond directly to their unique needs [14].

Meaningful engagement with communities also extends to contexts in which they are likely to interact with health interventions and environmental factors that contribute to the effectiveness of these systems. Developing interventions within the community context can foster awareness of the reality of how communities experience and interact with technology. For example, Muñoz and Arriaga [63] documented the preferences of low-income parents when tracking child development by using technology. In this work, the researchers met parents at centers for women, infants, and children and identified context-driven guidelines for technologies, including sharing information between multiple caregivers and across generations. Modifying studies to be culturally aware can foster greater participation from communities. From the same work by Muñoz and Arriaga [63], 1 member of this research team spoke Spanish, the dominant language in this community, and the researchers included Spanish materials. This led to a substantial increase in the recruitment of Spanish-speaking parents (nearly doubled). Researchers have also demonstrated that deploying interventions in diverse contexts requires attention to the unique community contexts. Escobedo and Arriaga [64] engaged with parents in a neighborhood childcare center, where they evaluated a milestone-tracking application. In this study, the researchers collaborated with Spanish-speaking parents and identified that official translations of developmental milestones from the Centers for Disease Control did not reflect the Spanish variant (Mexican Spanish), which is primarily spoken in the United States. Through careful engagement with communities, both design and evaluation processes can be responsive to the unique experiences of diverse communities.

Researchers might also engage families as designers of technologies to identify well-suited delivery methods and feature specifications. Studies have engaged families in design practice and have found that systems are better aligned with family experience [65]. The user interface and experience can also be honed through this type of research engagement [66]. Although this systematic review did not specifically focus on the design and evaluation of features in these technologies, researchers have demonstrated the influence of features on outcomes [67]. Although none of the articles included in this review included feature-level analyses, including the evaluation of features may lead to an understanding of what features affect proximal outcomes.

### Limitations

There are limitations to our findings. We did not include articles that described the components of an mHealth technology or a study to evaluate it but did not have participant groups using the technology (eg, study protocols). We also did not include studies where mHealth technology was a part of a larger intervention or studies of technologies developed for parents of children with specific health conditions, such as autism. This may exclude technologies that address areas of early childhood health promotion, specifically those covering developmental delays. Finally, our analysis of this work was heavily informed by Bright Futures Guidelines for Health Supervision of Infants, Children, and Adolescents, which was developed in the United States and thus could include content that is culturally different from developmental screening content in other countries. The Bright Futures guidelines are unique to the developmental screening processes in the United States, which may frame child health needs differently than other countries. As such, our analysis may not reflect each unique context in which these child health technologies have been developed.

### Conclusions

We conducted a systematic review of mobile-based technologies for the promotion of early childhood health. We categorized studies by field to identify trends in design and evaluation practices and opportunities for those processes to address health disparity reduction. More mHealth interventions are needed that comprehensively address all areas of early childhood health, including anticipatory guidance, development and behavior screening, and the social determinants of health screening. None of the studies evaluated in this review contributed to a system that addressed all 3 of these topics. To fully understand the accuracy of health recommendations and identify reasons for a lack of adherence, it is necessary for early childhood health promotion tools to comprehensively address all the areas affecting child health. Without considerations of upstream factors, intervention risk is less effective, particularly in underserved populations.

## Appendix

Multimedia Appendix 1 Full search strings by database.

## Abbreviations

mHealth: mobile health
PRISMA: Preferred Reporting Items for Systematic Reviews and Meta-Analyses
