# Supplementary material for: mHealth Technology Design and Evaluation for Early Childhood Health Promotion: Systematic Literature Review
Source: JMIR Pediatr Parent. 2022 Oct 6;5(4):e37718. doi: 10.2196/37718 (PMC9585442; doi:10.2196/37718)
Supplement: Multimedia Appendix 1 [file pediatrics_v5i4e37718_app1.docx]

### Multimedia Appendix 1 - Full Search Strings by Database

#### PubMed

((eHealth OR "mHealth" OR "mobile health" OR "health promotion" OR "health intervention") AND (mobile phone OR cell phone OR mobile application OR text messaging OR short message service OR smartphone) AND (developmental screening OR developmental milestone OR developmental checklist OR developmental progress OR "ESPDT" OR developmental tracking OR child health OR child development OR "physical well-being" OR "social and emotional development" OR "language development" OR "early literacy")) AND ("infant"[MeSH Terms] OR "infant"[MeSH Terms] OR "infant, newborn"[MeSH Terms] OR "child, preschool"[MeSH Terms]) AND ("Parents"[Mesh] OR "Legal Guardians"[Mesh] ) AND ("2011"[PDat] : "2022"[PDat])

#### EMBASE

('ehealth'/exp OR ehealth OR 'mhealth'/exp OR 'mhealth' OR 'mobile health'/exp OR 'mobile health' OR 'health promotion'/exp OR 'health promotion' OR 'health intervention') AND (((((mobile AND phone OR 'cell'/exp OR cell) AND phone OR mobile) AND ('application'/exp OR application) OR text) AND messaging OR short) AND message AND service OR 'smartphone'/exp OR smartphone) AND (((((developmental AND ('screening'/exp OR screening) OR developmental) AND milestone OR developmental) AND ('checklist'/exp OR checklist) OR 'espdt' OR developmental) AND tracking OR 'child'/exp OR child) AND ('health'/exp OR health) OR 'child'/exp OR child) AND ('development'/exp OR development OR 'developmental progress') AND ([newborn]/lim OR [infant]/lim OR [child]/lim OR [preschool]/lim) AND [2011-2022]/py

#### CINAHL Complete

((eHealth OR “mHealth” OR “mobile health”) AND (mobile phone OR cell phone OR mobile application OR text messaging OR short message service OR smartphone) AND (developmental screening OR developmental milestone OR developmental checklist OR “ESPDT” OR developmental tracking OR developmental progress OR child health OR child development) AND (infant OR newborn OR child, preschool) AND (parents OR mothers OR fathers OR legal guardian) AND (“health promotion” OR “health intervention”))

**Limiters (must be selected manually): Published Date: Start Month and Year: January 2011, End Month and Year: December 2022

#### ERIC

((“eHealth” OR “mHealth” OR “mobile health” OR “health intervention” OR "health promotion" Or “mobile health program”) AND (Telecommunications OR Handheld Devices OR Synchronous Communication OR “short message service” OR “text messaging” OR “smartphone” OR “mobile phone” OR “mobile app”) AND (“developmental screening” OR “developmental milestone” OR “developmental checklist” OR “ESPDT” OR “developmental tracking” OR “Child Development” OR “Child Health” OR “Developmental Stages”) AND (Infants OR newborn OR Preschool Children OR Young Children OR Toddlers) AND (Parents OR mothers OR fathers OR legal guardian) NOT (Developmental Delays OR Neurological Impairments OR Disability OR Autism OR “mental disorder”) NOT (Adolescents OR Teenagers OR Preadolescents) NOT ("education" OR “school” OR "child abuse") ) **Filter: last 10 years

#### Compendex and Inspec (Using Engineering Village)

((((((mobile phone OR mobile app OR smartphone OR phone app OR texting OR text messaging OR SMS OR mobile health OR mhealth OR eHealth OR "health promotion" OR "health intervention" OR "public health")) WN ALL) AND ((("developmental milestones" OR "developmental progress" OR "developmental tracking" OR "child development" OR "child health outcomes" OR "developmental stages" OR "developmental screening" OR "developmental checklists" OR "ESPDT")) WN ALL)) AND (((child OR infant OR newborn OR preschool child OR young children OR toddlers)) WN ALL)) NOT ((("pregnant women")) WN ALL)) **Filter: “Published” field to 2011-2022

#### ACM Digital Library

(ehealth OR "mhealth" OR "mobile health" OR "health promotion" OR "health intervention") AND (mobile phone OR cell phone OR mobile application OR text messaging OR short message service OR smartphone) AND (developmental screening OR developmental milestone OR developmental checklist OR "espdt" OR developmental tracking OR developmental progress OR child health OR child development) AND (infant OR newborn OR child, preschool) AND (parent OR mother OR father OR legal guardian)

**Filter: “Publication Date” field: January 2011 - December 2022
